# Background inhibited and speed-loss-free volumetric imaging *in vivo* based on structured-illumination Fourier light field microscopy

**DOI:** 10.3389/fnins.2022.1004228

**Published:** 2022-09-29

**Authors:** Jiazhen Zhai, Ruheng Shi, Kuikui Fan, Lingjie Kong

**Affiliations:** ^1^State Key Laboratory of Precision Measurement Technology and Instruments, Department of Precision Instrument, Tsinghua University, Beijing, China; ^2^IDG/McGovern Institute for Brain Research, Tsinghua University, Beijing, China

**Keywords:** Fourier light field microscopy, structured-illumination, large-scale imaging, high resolution imaging, fast volumetric imaging, background inhibited, speed-loss-free

## Abstract

Benefiting from its advantages in fast volumetric imaging for recording biodynamics, Fourier light field microscopy (FLFM) has a wide range of applications in biomedical research, especially in neuroscience. However, the imaging quality of the FLFM is always deteriorated by both the out-of-focus background and the strong scattering in biological samples. Here we propose a structured-illumination and interleaved-reconstruction based Fourier light field microscopy (SI-FLFM), in which we can filter out the background fluorescence in FLFM without sacrificing imaging speed. We demonstrate the superiority of our SI-FLFM in high-speed, background-inhibited volumetric imaging of various biodynamics in larval zebrafish and mice *in vivo*. The signal-to-background ratio (SBR) is improved by tens of times. And the volumetric imaging speed can be up to 40 Hz, avoiding artifacts caused by temporal under-sampling in conventional structured illumination microscopy. These suggest that our SI-FLFM is suitable for applications of weak fluorescence signals but high imaging speed requirements.

## Introduction

Fast, high quality volumetric imaging facilities many attractive applications in the biomedical studies, such as the large-scale neural activity imaging (Demas et al., 2021; Zhang et al., 2021c), hemodynamic imaging (Wagner et al., 2019; Wagner et al., 2021), neurovascular coupling imaging (Park et al., 2017; Fan et al., 2020), and the cytology research (Hua et al., 2021; Wu et al., 2021), etc. The urgent need for high-speed volumetric imaging has spawned the development of various techniques, such as the multiphoton fluorescence microscopy (Zipfel et al., 2003), the confocal microscopy (Jonkman et al., 2020), and the light sheet microscopy (Bouchard et al., 2015; Voleti et al., 2019), etc. These methods have shown excellent performance and have been commercialized and have advanced biomedical research for years. However, the imaging speed of conventional volumetric imaging microscopy is limited either by its mechanical scanning speed or the detector response speed (Jin et al., 2020; Chang et al., 2021). This makes them typically only possible to image several planes with specific depths at lower speed. Besides, these methods usually rely on complex systems. For example, two-photon microscopy requires expensive lasers and complex scanning systems, and light sheet microscopy requires multiple objectives, etc.

On the contrary, the recently developed light field microscopy (LFM) has shown great advantages in fast volumetric imaging (Levoy et al., 2006; Broxton et al., 2013; Prevedel et al., 2014; Wagner et al., 2021; Wu et al., 2021). Its super volumetric imaging capability has been used in a variety of applications (Prevedel et al., 2014; Pégard et al., 2016; Li et al., 2019; Chen et al., 2020; Sims et al., 2020; Xiong et al., 2021; Zhang et al., 2021a,b), especially in neuroscience research *in vivo*. In recent years, LFM has developed rapidly. Among the various developments of LFM (Levoy et al., 2006; Nobauer et al., 2017; Guo et al., 2019; Li et al., 2019; Stefanoiu et al., 2019; Cai et al., 2020; Chen et al., 2020; Wagner et al., 2021; Wang D. et al., 2021; Wang Z. et al., 2021; Wu et al., 2021), the Fourier light field microscopy (FLFM) (Llavador et al., 2016; Cong et al., 2017; Guo et al., 2019; Yanny et al., 2020; Yoon et al., 2020; Hua et al., 2021) has unique advantages. By performing the multi-views imaging simultaneously, FLFM can reconstruct a volumetric image with a single exposure, pushing the volumetric imaging speed up to the limit of the camera. More importantly, FLFM takes advantage of its uniform point spread function (PSF) (Cong et al., 2017; Guo et al., 2019), which helps FLFM to avoid the severe reconstruction artifacts of LFM near the focal plane (Broxton et al., 2013; Wagner et al., 2021). Benefiting from the advantages, FLFM has been widely used in many applications, achieving large field-of-view (FOV) (Xue et al., 2020) and depth-of-field (DOF) (Cong et al., 2017; Yoon et al., 2020) with a relatively high resolution (Hua et al., 2021; Liu and Jia, 2021).

However, in the *in vivo* imaging applications, the FLFM still suffers from strong out-of-focus signals and tissue scattering (Zhang et al., 2021a,c; Zhai et al., 2022). The former is due to the inherent wide-field illumination methods, while the latter is caused by the non-uniform distribution of tissues. These issues not only deteriorate imaging quality, but also increase the burden of reconstruction (Yoon et al., 2020). So far, various techniques have been proposed. Inspired by previous optical sectioning techniques, researchers have integrated the selective-volume-illumination methods [such as confocal illumination (Zhang et al., 2021c), two-photon excitation (Madaan et al., 2021), light-sheet illumination (Wang et al., 2019; Wang Z. et al., 2021), structured illumination (Taylor et al., 2018; Fu et al., 2021), etc.] and the selective-volume-detection methods [such as the computational methods (Zhang et al., 2021a), confocal slit detection (Zhang et al., 2021c), etc.] into the FLFM. These methods have successfully improved the image quality of LFM. However, the former fails in avoiding the tissue scattering physically, and the latter methods require prior assumptions of samples or complex system design.

To overcome the shortcomings of above methods, we have recently proposed the robust Fourier light field microscopy (RFLFM) (Zhai et al., 2022). By introducing the “HiLo” structured illumination and computational reconstruction (Lim et al., 2008; Santos et al., 2009; Lim et al., 2011), we can remove the background signals and the tissue scattered light simultaneously by post-processing. Moreover, it is worth noting that the RFLFM can be easily adopted by adding a deformable mirror device (DMD) in the illumination path of FLFM. However, the main disadvantage of RFLFM is the a-half loss of imaging speed. Same as the optical-sectioning wide field microscopy (Lim et al., 2008; Santos et al., 2009; Lim et al., 2011; Shi et al., 2019), RFLFM needs to take a structured illumination (SI) image and a uniform illumination (UI) image together to recover an optical-sectioning image. This, unfortunately, decreases the imaging speed and increases the storage burden.

Another computed tomography-based microscopy, the structured illumination microscopy (SIM) (Bozinovic et al., 2008; Mertz, 2011; Hagen et al., 2012; Dongli et al., 2013; Zhou et al., 2015), is also widely used. SIM recovers an optical-sectioning image by sequentially changing the phase of the structured illumination patterns with $\frac{2\,\pi }{3}$. Therefore, the traditional SIM needs to take three consecutive images to recover a single optical sectioned image, which is even slower than HiLo. However, inspired from the periodicity of the sine function, SIM has the potential to restore the original imaging speed (Shi et al., 2021). When we switch the SIM illumination patterns continually, the phases of all patterns are physically continuous too. Thus, we can extract new structured illumination periods from the original periods to make up the speed loss.

Here, we propose the structured-illumination and interleaved-reconstruction based Fourier light field microscopy (SI-FLFM). By using SI-FLFM, we can eliminate the background fluorescence in Fourier light field imaging without decreasing imaging speed. We demonstrate the superiority of our SI-FLFM in high-speed, background-inhibited volumetric imaging of various biodynamics in both larva zebrafish and mice *in vivo*. The results show that our system has achieved great improvements in both imaging quality and imaging speed, which will facilitate the wide application of our SI-FLFM in biomedical research.

## Materials and methods

### Optical design

**FIGURE 1:**
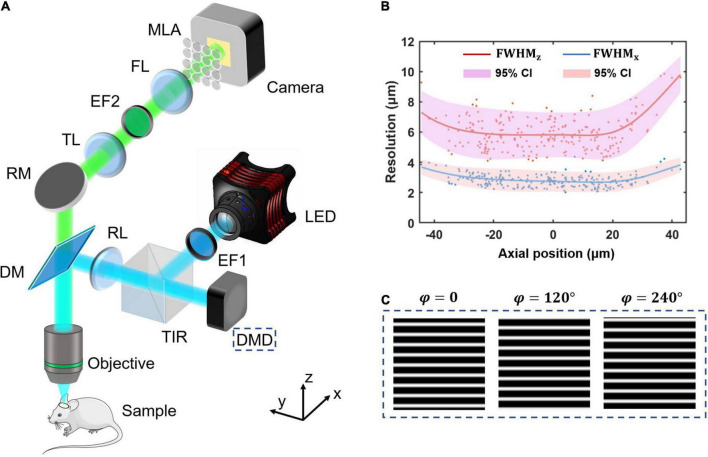
The system design and resolution calibration of SI-FLFM. **(A)** Optical scheme of SI-FLFM. The digital micromirror device (DMD) plane is conjugated with the native objective plane (NOP), thus projects the structured patterns on the sample. EF1, excitation filter 1; DMD, digital micromirror device; TIR, total internal reflection prism; RL, relay lens; DM, dichroic mirror; RM, reflector mirror; TL, tube lens; EF2, emission filter 2; FL, Fourier lens; MLA: microlens array. **(B)** Three-dimensional resolution with 95% confidence interval (CI), fitted by LOESS algorithm. The resolution is calibrated by full-width-at-half-maximum (FWHM) of the imaging results of sub-resolution micro-beads (Φ = 1.1 μm). **(C)** The generated sinusoidal patterns of different phases for illumination are loaded in the memory of DMD. The fringe period is 90 pixels, calculated based on the DOF of SI-FLFM.

Structured-illumination Fourier light field microscopy is built by introducing structured illumination in the conventional FLFM. We firstly design a FLFM for biological imaging *in vivo*, and then introduce a digital micromirror device (DMD) into its illumination path to project the structured patterns. The key of designing FLFM is to assign spatial spectrum information to different microlens, resulting in multi-view imaging. And the three-dimensional (3D) reconstruction is carried out through the Richardson-Lucy deconvolution algorithm. Figure 1 shows the system design (same as that in our former report (Zhai et al., 2022)) and resolution calibration of the SI-FLFM.

In the illumination path, we choose a collimated LED (SOLIS-470C, Thorlabs) for high-power excitation. The excitation light is filtered by the EF1 (Excitation Bandpass Filter, MF469-35, Thorlabs) to narrow the spectrum with a center wavelength at λ_*ex*_≈470nm, followed by being reflected to the DMD (1920 × 1080 pixels, DLP9500, TI) by a total internal reflection prism (TIR). The DMD consists of a micro-mirror array, in which each mirror can be mechanically rotated to achieve binarized projection. A dichroic mirror (DM, DMLP490, Thorlabs) is used to separate excitation and emission light. We then use two 4*f* relay systems, the first consisting of two doublets lens (*f* = 150 mm, AC508-150A and *f* = 300 mm, AC508-300A, Thorlabs, not shown in Figure 1A) and the second consisting of an RL (Relay Lens, *f* = 200 mm, AC508-200A, Thorlabs) and an objective (*f* = 7.2 mm, numerical aperture is 1.05, XLPLN25XWMP2, OLYMPUS). Thus, the surface of the DMD is conjugated to the native object plane (NOP) of the objective. When we load structured patterns of different phases (as shown in Figure 1C) on the DMD, they will modulate the in-focus sample in the object space correspondingly. The illumination path is designed as *Köhler Illumination* to perform uniform illumination.

In the detection path, the emission light from the sample is collected by the objective and then imaged by a tube lens (TL, *f* = 200 mm, AC508-200A, Thorlabs) at 27.78 times magnification. On the back focal plane of TL, we place an emission bandpass filter (EF2, customized, λ_*em*_≈525*nm*, Edmund) to filter out the residual excitation light. We then use a Fourier lens (FL, *f* = 300 mm, AC508-300A, Thorlabs) to perform optical Fourier transform. On the back focal plane of FL, a microlens array (MLA, FEL-46S03-38.24PM, *d* = 3 mm, *f* = 38.24 mm, Sigma) is placed. Different microlens intercepts spatial spectra differently, performing the multi-view imaging on the camera (S-25A80 CoaXPress, Adimec).

**FIGURE 2:**
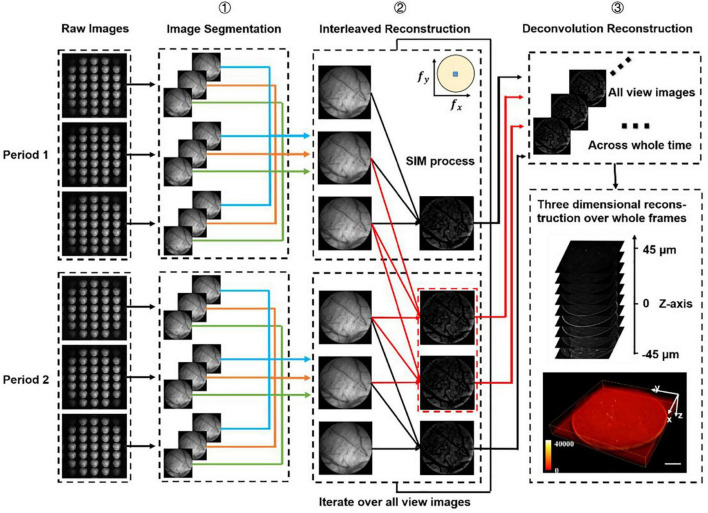
Data processing procedure. We firstly divide all the raw images (number: 3*n*) into different periods (number: *n*), and we have chosen two periods as an example to demonstrate the processing procedure. Step 1: Segment all raw images into sub-images at multi-views. Step 2: Use optical-sectioning algorithms and interleaved reconstruction to achieve optical-sectioning (OS) images without losing frames. The red lines indicate the adding frames recovered by interleaved reconstruction. The number of OS images is 3*n*-2 rather than *n* for each view. This step will iterate until all views sub-images have been processed. Step 3: 3D reconstruction for whole frames by the Richardson-Lucy deconvolution algorithm.

Based on the optical design above, our system works at 3.54 times magnification and 0.139 NA for all 31 views (as shown in Figure 2) imaging. The maximum field of views (FOV) is up to Φ = 840 μm, the depth-of-field (DOF) is about 90 μm. The size of the surface of the DMD after being projected by the illumination light path is 1,493 μm × 840 μm, which can cover the entire FOV.

### System calibration

To demonstrate that our system can work at cellular resolution, we experimentally calibrate the resolution by imaging fluorescent beads (Φ = 1.1 μm, Thermo Fisher Scientific).

Due to the inevitable aberration in the system, it is difficult to achieve accurate reconstruction with the simulated point-spread-function (PSF) (as shown in Supplementary Figure 1a). Thus, we firstly calibrate the real PSF of the system by imaging the sparse fluorescence beads. To prepare the fluorescent beads sample for calibration, we dilute the original fluorescent beads solution by 2.5 × 10^5^ times in agar at 95°C, then take a little of the solution on the glass slide and cool it to room temperature. The sample is sparse enough that only about 1 bead can be found in the whole FOV. After placing the sample, we move the stage (MT3/M-Z8, Thorlabs) at 1.5 μm steps and record the imaging results over a 90 μm range centered on the NOP. Based on these raw images, we obtain the PSFs of all views across the whole depth range (as shown in Supplementary Figure 1b) through the maximum connected domain extraction algorithm in MATLAB.

To calibrate the 3D resolution of the system, we then configure a 1:1000 dilution of fluorescent beads (Φ = 1.1 μm, Thermo Fisher Scientific) as sample. Through a single exposure of the sample, we obtain the raw image of a large number of fluorescent beads distributed in 3D space. Based on the previously acquired center coordinates of the sub-images for each view, we segment the original image to 31 individual images of different views. Then we use the extracted real PSFs and Richardson-Lucy algorithm to reconstruct the 3D image. We use the FWHM (full-width-at-half-maximum-intensity) to represent the resolution. However, off-axis aberrations (such as coma) distributed elsewhere in the FOV still can’t be avoided even with the real PSFs. Therefore, different locations in the FOV may show different resolutions. We use the Local Weighted Linear Regression (LOESS) algorithm (Hua et al., 2021) to fit the resolutions. Based on the linear least squares and first-degree polynomial model, the LOESS algorithm can be addressed by solving the optimization problem:


(1) $$m\,i\,n_{\left(\alpha \,\left(x_{0}\right),\beta \,\left(x_{0}\right)\right)}\,F\,\left(\alpha \,\left(x_{0}\right),\beta \,\left(x_{0}\right)\right)$$

(2) $$F\,\left(\alpha \,\left(x_{0}\right),\beta \,\left(x_{0}\right)\right)=\sum_{i=1}^{N}K_{d}\,\left(x_{0},x_{i}\right)\,{\left[y_{i}-\alpha \,\left(x_{0}\right)-\beta \,\left(x_{0}\right)\,x_{i}\right]}^{2}$$

Where *K*_*d*_(*x*_0_,*x*_*i*_) is the weighting factor determined by the distance from *x_i_* to *x_0_*.


(3) $$K_{d}\,\left(x_{0},x_{i}\right)=e\,x\,p\,\left(-\frac{{\left(x_{0}-x_{i}\right)}^{2}}{2\,d^{2}}\right)$$

Here, we set the *d* to be 10 μm. The predicted resolution at *x_0_* is $y_{0}=\hat{\alpha }\,\left(x_{0}\right)+\hat{\beta }\,\left(x_{0}\right)\,x_{0}$. Based on the normal distribution, two standard deviations of the fit represent the 95% confidence interval. So, we calculate the standard deviation as:


(4) $$\sigma_{x_{0}}=\sqrt{\frac{F\,\left(\alpha \,\left(x_{0}\right),\beta \,\left(x_{0}\right)\right)}{\sum_{i=1}^{N}K_{d}\,\left(x_{0},x_{i}\right)}}$$

Thus, the 95% confidence interval at *x_0_* is from *y*_0_−2_σ*x*_0__ to *y*_0_ + 2_σ*x*_0__. As shown in Figure 1B, the lateral resolution varies in 2∼4 μm as well as the axial resolution varies in 4∼10 μm in a 90 μm range DOF. The results suggest that our system works at cellular resolution.

### System synchronization

Structured-illumination Fourier light field microscopy needs accurate system synchronization to perform structured illumination, which is achieved with a microcontroller (UNO Rev3, Arduino). As shown in Supplementary Figure 2, the microcontroller generates the digital signals, which are transmitted to the camera and DMD simultaneously. The camera starts an exposure once receiving a high-level signal (“1”), which will last for the same duration as that of the high-level signal. Meanwhile, the DMD will refresh the pattern to the next one after receiving a high-level signal. The refresh is so fast (about 20,000 Hz) that the settle time can be ignored. After the exposure, the camera needs about 12 ms to read out the image to RAM. Thus, the microcontroller is settled to a low level (“0”) signal for 12 ms to finish the transmission. In this way, we achieve a raw image under structured illumination.

### Principles of structured-illumination Fourier light field microscopy and data processing procedures

In SI-FLFM, we need to integrate the optical sectioning algorithm and the interleaved reconstruction algorithm into the Richardson-Lucy deconvolution. Here, we demonstrate the data processing procedure of two structure illumination periods in Figure 2.

### Optical sectioning algorithm

We use *I*_1_,*I*_2_, and *I*_*3*_ to represent the images at different phases in a structured illumination period. Based on the modulating properties, the *I*_1_,*I*_2_ and *I*_*3*_ can be expressed by:


(5) $$\left\{ \begin{array}{c} I_{1}=I_{o\,u\,t-o\,f-f\,o\,c\,u\,s}+I_{i\,n-f\,o\,c\,u\,s}\,\sin\left(M\,x+\varphi \right)\\ I_{2}=I_{o\,u\,t-o\,f-f\,o\,c\,u\,s}+I_{i\,n-f\,o\,c\,u\,s}\,\sin\left(M\,x+\varphi +\frac{2}{3}\,\pi \right)\\ I_{3}=I_{o\,u\,t-o\,f-f\,o\,c\,u\,s}+I_{i\,n-f\,o\,c\,u\,s}\,\sin\left(M\,x+\varphi +\frac{4}{3}\,\pi \right) \end{array}\right.$$

M is the modulation period and is constant. Thus, the optical-sectioning (OS) images can be calculated by:


(6) $$I_{O\,S}=I_{i\,n-f\,o\,c\,u\,s}=\sqrt{{\left(I_{1}-I_{2}\right)}^{2}+{\left(I_{1}-I_{3}\right)}^{2}+{\left(I_{2}-I_{3}\right)}^{2}}$$

However, since we use DMD for structured patterns projection, there will be periodic fringe artifacts in the reconstructed images. Moreover, the square operation will turn the negative signal in the subtraction into a positive value, thereby amplifying the high-frequency noise and reducing the signal-to-noise ratio (SNR) (Li et al., 2020). This problem can be solved by the refined SIM (RSIM) (Zhou et al., 2015). We can firstly get the uniform illumination (UI) images by:


(7) $$I_{U\,I}=I_{i\,n-f\,o\,c\,u\,s}+I_{o\,u\,t-o\,f-f\,o\,c\,u\,s}=\frac{I_{1}+I_{2}+I_{3}}{3}$$

The *I*_*out–of–focus*_ only contributes the low-frequency information in the *I*_*UI*_, but the *I*_*in–focus*_ has both low-frequency and high-frequency information. Therefore, we can apply a high-pass filter on *I*_*UI*_ to obtain the high-frequency part of the *I*_*in–focus*_:


(8) $$H\,P\,\left(I_{i\,n-f\,o\,c\,u\,s}\right)=H\,P\,\left(I_{U\,I}\right)$$

The *I*_*OS*_ contains only in-focus information with periodic fringes artifacts. However, the periodic fringe artifacts are high frequency noise and we can filter it out using low pass filtering:


(9) $$L\,P\,\left(I_{i\,n-f\,o\,c\,u\,s}\right)=L\,P\,\left(I_{O\,S}\right)$$

Finally, we can extract the in-focus information with full frequency:


(10) $$I_{i\,n-f\,o\,c\,u\,s}=L\,P\,\left(I_{i\,n-f\,o\,c\,u\,s}\right)+H\,P\,\left(I_{i\,n-f\,o\,c\,u\,s}\right)=L\,P\,\left(I_{O\,S}\right)+H\,P\,\left(I_{U\,I}\right)$$

### Interleaved reconstruction

Same as above, *I*_1_,*I*_2_ and *I*_*3*_ are used to represent the images at different phases in structured illumination period 1, and *I*_4_,*I*_5_ and *I*_*6*_ are those for period 2. We take *I*_*1*_ to *I*_*6*_ at different times from t_1_ to t_6_. Using the periodicity of trigonometric functions, they can be expressed as Equation (5) and:


(11) $$\left\{ \begin{array}{c} I_{4}=I_{o\,u\,t-o\,f-f\,o\,c\,u\,s}+I_{i\,n-f\,o\,c\,u\,s}\,\sin\left(M\,x+\varphi +2\,\pi \right)\\ I_{5}=I_{o\,u\,t-o\,f-f\,o\,c\,u\,s}+I_{i\,n-f\,o\,c\,u\,s}\,\sin\left(M\,x+\varphi +\frac{8}{3}\,\pi \right)\\ I_{6}=I_{o\,u\,t-o\,f-f\,o\,c\,u\,s}+I_{i\,n-f\,o\,c\,u\,s}\,\sin\left(M\,x+\varphi +\frac{10}{3}\,\pi \right) \end{array}\right.$$

We can extract the in-focus information at t_2_ and t_5_ through the optical sectioning algorithm. However, we can find that *I*_2_,*I*_3_ and *I*_*4*_ and *I*_3_,*I*_4_ and *I*_*5*_ also satisfy the conditions of the algorithm. Thus, we can extract two new periods and two new optical-sectioning images at t_3_ and t_4_. If we have 3*n* raw images, through the interleaved reconstruction method, we can extract 3*n*-2 optical-sectioning images instead of *n*.

#### Data processing procedures

All the procedures of data processing are shown in Figure 2. We first classify all raw images (number: 3*n*) according to the period of structured illumination (number: *n*), with each period containing three raw images of different phases. Then we split all raw images into sub-images of different views (number: 3 × 31 × *n*). We use optical-sectioning algorithms and interleaved reconstruction to extract optical-sectioning images for each group of sub-images. We repeat this process until all groups of sub-images are processed. At this point, we have 31 × (3*n*-2) sub-images. Finally, we use the real PSF and Richardson-Lucy deconvolution for 3D reconstruction.

### Animals

All procedures involved animals have been approved by the Institutional Animal Care Use Committee (IACUC) of Tsinghua University.

Rasgrf-dCre: Ai148 mice (8–9 weeks old) obtained from Charles River Laboratories (Beijing, China) are used in biological experiments. All mice are maintained in a temperature-controlled room on a 12-h light/dark cycle (lights on 06:00–18:00) with *ad libitum* access to food and water. Trimethoprim (TMP)-inducible Cre mice (dCre) receive one gavage dosing of TMP (0.3 mg/g body weight) per day for 1–3 days to active the Cre recombinase.

The zebrafish larvae (5–7 days old) expressing GCaMP6f (Tg (HUC: H2B-GCaMP6f)) are used in the neural network imaging experiment. And the hematopoietic stem cells labeled zebrafish larvae [Tg (runx1: GFP)] at 2–4 days’ are used in the hemodynamic imaging experiment.

### Craniotomy

Mice are under anesthetization with isoflurane and the body temperature is maintained at 37°C. Mouse eyes are covered with ophthalmic ointment to prevent drying and the mice are mounted in a stereotaxic frame with ear bars. A surgical area with an electric shave is cleared. We first cut and remove the skin over the skull surface and remove the periosteum to expose and clean the surface of the skull. Next, we carefully open an 8 mm × 8 mm diameter window on the skull using a miniature hand-held skull drill (68605, RWD Life Science). Furthermore, we use PBS buffer to clean blood stains, and a round glass sheet is placed on the window. We fix the glass window with glue and add an aluminum fixing pole. The rest of the skull surface is covered as well, making sure the edges of the skin are covered by cement and letting it dry. Following the surgery, the mice are left in a cage until they fully recover. We inject 5% (w/v) glucose in saline (s.c.) for rehydration and 0.05 to 0.1 mg/kg buprenorphine (i.p., instant release) in mice for post-operative analgesia. To minimize the potential immunological reaction, the mice are i.p. injected daily with 20 μl/100 g cyclosporine.

## Experimental results

### Structured-illumination Fourier light field microscopy improves the signal-to-background ratio and effective resolution in neural network imaging of larval zebrafish *in vivo*

Zebrafish larvae are one of the commonly used samples for neuroscience research (Prevedel et al., 2014; Zhang et al., 2021b). To demonstrate the performance of SI-FLFM in removing background fluorescence, we perform *in vivo* imaging of neural activities in zebrafish larvae. We embed 5–7 days old zebrafish larvae expressing GCaMP6f [Tg (HUC: H2B-GCaMP6f)] in 1% agarose for the experiment. The volumetric rate of imaging is set as 10 Hz, and the volume center is about tens of microns below the surface of the larval zebrafish.

**FIGURE 3:**
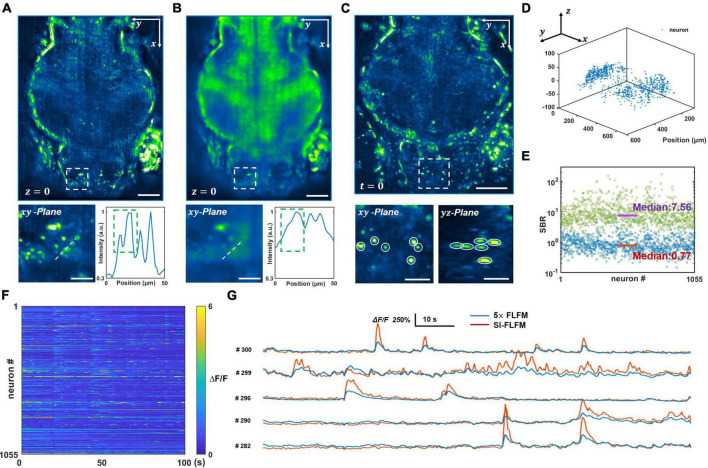
Volumetric imaging of neural network activity in the brains of larval zebrafish *in vivo*. **(A,B)** Maximum intensity projects (MIPs) over recorded time in the plane at *z* = 0, processed in SI-FLFM and FLFM modes, respectively. Scale bar: 100 μm in main figures and 30 μm in insets. **(C)** 3D view images of neurons in larval zebrafish, MIP over *z*-axis at *t* = 0, processed in SI-FLFM mode. Scale bar: 100 μm in main figures and 30 μm in insets. **(D)** 3D positions of flashing neurons. **(E)** Statistics of SBR over all neurons in both SI-FLFM (Green dots) and FLFM (Blue dots) modes. The median values are 7.56 and 0.77 in SI-FLFM and FLFM modes, respectively. **(F)** Fluorescence signal maps of flashing neurons in SI-FLFM mode. **(G)** Comparison of fluorescence signals of selected neurons between SI-FLFM and FLFM mode. The latter is magnified by a factor of 5.

We process both the optical-sectioning images from SI-FLFM and the superimposed images that are equivalent to uniform illumination situation in conventional FLFM. The same procedure will be performed below without specific clarification. We reconstruct a DOF of 90 μm and show the processed results of SI-FLFM and FLFM on the *z* = 0 plane in Figures 3A,B, respectively. It can be seen that two adjacent neurons are almost indistinguishable due to the interference from background fluorescence in the FLFM image. In contrast, we can clearly distinguish different neurons in the image of SI-FLFM. The 3D view images of neurons at *t* = 0 are shown in Figure 3C. It indicates that SI-FLFM greatly improves the effective resolution of the system (Yoon et al., 2020; Zhai et al., 2022).

Moreover, we extract the calcium signal of neural activities in the results of SI-FLFM by CNMF-e algorithm (Zhou et al., 2018). We extract five planes separated by 20 μm in axial direction, and find a total of 1,055 flashing neurons. The positions of all 1,055 neurons are labeled in Figure 3D, which indicates that neurons distribute in the whole volume. And the fluorescence signal maps are shown in Figure 3F. According to the positions, we extract the corresponding neurons from the FLFM images, and take statistics of their SBRs separately. As shown in Figure 3E, the median values of the SBRs are 7.56 and 0.77 in SI-FLFM and FLFM modes, respectively. This means that the SBR is improved by about 10 times in SI-FLFM without reducing imaging speed. To more intuitively compare the neural activity imaging results in these two modes, we pick 5 neurons and compare their signal trajectories in Figure 3G. We can see that the signal fluctuations of SI-FLFM obviously exceeds that of FLFM by five times. The results suggest that SI-FLFM can effectively remove the fluorescence background which exists in conventional FLFM and enhance the effective resolution of the system.

### Structured-illumination Fourier light field microscopy achieves blood cell tracking in larval zebrafish *in vivo* with high signal-to-background ratio

The study of hemodynamics relies on high-speed, high-SBR volumetric imaging (Wagner et al., 2019; Wagner et al., 2021), where SI-FLFM is an ideal tool. We demonstrate the unique advantages of SI-FLFM in 3D tracking of blood cells in larval zebrafish [Tg (runx1: GFP)] *in vivo*. The volumetric rate of imaging is set up to 40 Hz, and the volume center is about tens of microns below the surface of the larval zebrafish.

**FIGURE 4:**
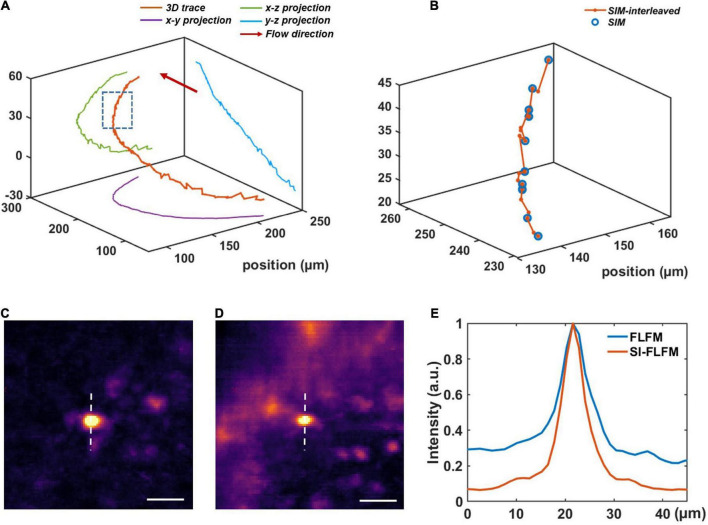
High-speed blood cell tracking in larval zebrafish *in vivo* with high SBR. **(A)** 3D trace of a single blood cell. **(B)** Enlarged view of the inset box in panel **(A)**. We can record more positions by using the interleaved reconstruction method. **(C,D)** Images of the tracked blood cell at *t* = 22.30 s in SI-FLFM and FLFM modes, respectively. Scale bar: 25 μm. **(E)** Comparison between the normalized intensity profiles of the dotted lines in Figures 5C,D.

We perform the volumetric reconstruction for a volume of Φ840 μm × 90 μm and manually track a blood cell as an example. The center positions at different times are obtained by calculating the centroid of the cell. Without interleaved reconstruction, the volumetric rate will be limited to 13.3 Hz, which is obviously insufficient for high-speed imaging of blood flow. However, in SI-FLFM, we can increase the effective volumetric rate to 40 Hz. As shown in Figures 4A,B, we can see that more positions are distinguished by using the interleaved reconstruction method. Besides, in the results of SI-FLFM, the SBR is significantly improved, compared with that in FLFM, which is shown in Figures 4C–E. The results show that SI-FLFM is suitable for high-speed 3D imaging with high SBR.

### Structured-illumination Fourier light field microscopy enables speed-loss-free, high-signal-to-background ratio functional imaging in mouse cortex *in vivo*

*In vivo* imaging of neural activity in mouse cortex is of great value in neuroscience research (Park et al., 2017; Zhang et al., 2021c; Zhai et al., 2022). However, it is extremely challenging for single-photon fluorescence imaging of mouse cortex, as it is often disturbed by scattering from the turbid tissue and the strong out-of-focus signals. Therefore, effectively suppressing the background is the key to overcome this problem. We demonstrate that SI-FLFM enables speed-loss-free and high SBR functional imaging in mouse cortex *in vivo*.

**FIGURE 5:**
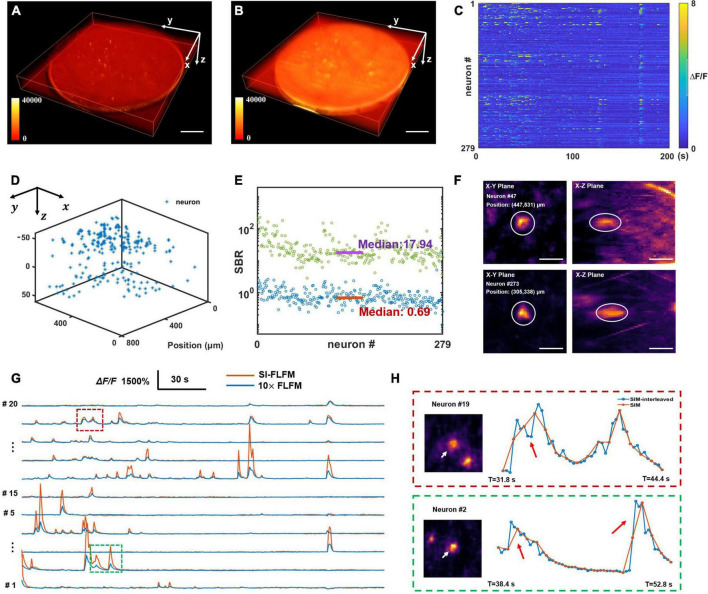
High-speed volumetric imaging of neural network activity in mouse brains *in vivo* with high SBR. **(A,B)** MIPs over time in the four-dimensional domain (*x-y-z-t*), processed in SI-FLFM and FLFM modes, respectively. Scale bar: 100 μm. **(C)** Fluorescence signal maps of detected neurons, based on the results in SI-FLFM mode. **(D)** 3D positions of detected neurons. **(E)** Statistic of SBR over all neurons in both SI-FLFM (Green dots) and FLFM (Blue dots) modes. The median values are 17.94 and 0.69 in SI-FLFM and FLFM modes, respectively. **(F)** 3D view images of neurons (neuron #47 and neuron #273) in mouse cortex after MIP over time. Scale bar: 20 μm. **(G)** Comparison of the selected neurons’ fluorescence signals in SI-FLFM and FLFM modes. The latter is magnified by a factor of 10. **(H)** Enlarged profiles of the inset boxes in panel **(G)**. The additional intensity traces help to avoid artifacts in functional imaging, such as the numbers (red box labeled) and the accurate times (green box labeled) of neuronal firings. Scale bar: 20 μm.

Firstly, we perform the craniotomy for the adult mice (Rasgrf-d Cre: Ai148), which sparsely express the GCaMP6f only in layer 2/3. After 2 weeks of recovery, we perform functional imaging when the mouse is awake and head-restrained under the microscope. Considering that prolonged high-intensity excitation can induce photobleaching or even cause neural death, the intensity of the excitation light has to be reduced. Meanwhile, we need to ensure sufficient exposure, so we set the volumetric rate as 5 Hz. And the volume center is about 150–200 microns below the surface of mouse dura. Both types of results are processed. As shown in Figures 5A,B, SI-FLFM can significantly filter out background fluorescence. We show the 3D view images of two neurons (neuron #47 and neuron #273) as examples in Figure 5F. We use the CNMF-e algorithm (Zhou et al., 2018) to perform calcium signal extraction on five planes spaced 20 μm apart in axial direction. We label the positions and the fluorescence signal maps of all 279 neurons in Figures 5C,D. And we calculate the correlation coefficients between the activity traces of each neuron. As shown in Supplementary Figure 3, the distribution of correlation coefficients show that we can extract undisturbed free-firing signals by using SI-FLFM.

In addition, to verify the improvement of SI-FLFM in terms of SBR and imaging speed. We perform statistics of the SBR for all neurons in the volume in processed results of both SI-FLFM and FLFM (as shown in Figure 5E). The median values are 17.94 and 0.69 in SI-FLFM and FLFM modes, respectively. This shows that we have effectively remove the background and greatly improve SBR. Besides, we compare the calcium traces of several neurons in the two modes (Figure 5G), and the results suggest that SI-FLFM can amplify the fluorescence fluctuations of neural activity signals, i.e., △*F*/*F*, more than 10-fold. In terms of imaging speed, we increase it from 1.67 to 5 Hz through the interleaved reconstruction. As shown in Figure 5H, the additional intensity results help to avoid artifacts caused by temporal under-sampling in functional imaging. For example, the number of neuron firings (neuron #19, marked by the red box) and the exact firing time (neuron #2, marked by the green box) can be recorded by SI-FLFM, which would be missed without the interleaved reconstruction.

## Discussions and conclusions

We propose the novel SI-FLFM, which can be easily modified by adding a DMD into the FLFM. We demonstrate that SI-FLFM is suitable for large-scale, high-speed and high-resolution 3D imaging without background interference.

Through Richardson-Lucy deconvolution using the real PSF, SI-FLFM can achieve lateral 2∼4 μm and axial 4∼10 μm resolution within a volume of Φ = 840 μm (FOV) × 90 μm (DOF), which is promising for large-scale high-resolution imaging of biodynamics. To demonstrate the superior performance of SI-FLFM in removing background fluorescence, we perform functional imaging of neural network activities in both larval zebrafish and mouse cortex *in vivo*. Compared with FLFM, SI-FLFM can improve the SBR of imaging by tens of times and significantly improves the effective resolution.

Furthermore, we use the physical characteristics of structured illumination to avoid the loss of imaging speed through the interleaved reconstruction. The volumetric rate is up to 40 Hz. In the larval zebrafish blood flow imaging, more motion positions can be tracked by SI-FLFM, which is beneficial to the study of hemodynamic. In the functional imaging of the mouse cortex, the number of neuron firings and the exact firing time can be recorded by SI-FLFM, benefiting from the interleaved reconstruction. These suggest that artifacts caused by temporal under-sampling can be avoided in SI-FLFM. The theoretical volumetric imaging speed of SI-FLFM can be up to 84 Hz, limited by the read-out time of the camera.

In conclusion, SI-FLFM has great advantages in high-speed, high-SBR volumetric imaging of biodynamics. We expect that SI-FLFM will facilitate wide applications in biomedical research.

## Data availability statement

The original contributions presented in this study are included in the article/Supplementary material, further inquiries can be directed to the corresponding author.

## Ethics statement

The animal study was reviewed and approved by the Institutional Animal Care Use Committee (IACUC) of Tsinghua University.

## Author contributions

LK conceived the idea of and supervised this project. JZ developed the methods. JZ and RS designed the data processing algorithms. JZ and KF performed the experiments. All authors contributed to the writing of the manuscript and agreed to the submission.
