# Supplementary material for: Background inhibited and speed-loss-free volumetric imaging in vivo based on structured-illumination Fourier light field microscopy
Source: Front Neurosci. 2022 Sep 29;16:1004228. doi: 10.3389/fnins.2022.1004228 (PMC9558295; doi:10.3389/fnins.2022.1004228)
Supplement: Supplementary file 1 [file Data_Sheet_1.pdf]

# Supplementary Information

## Background inhibited and speed-loss-free volumetric imaging *in vivo* based on structured-illumination Fourier light field microscopy

# System Calibration

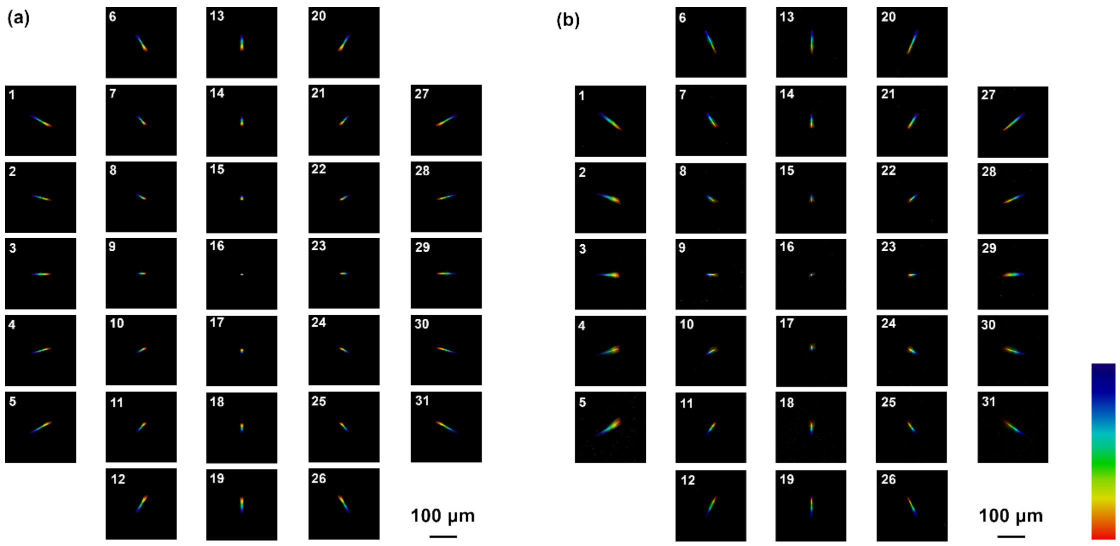


Fig. S1. The simulated and real recorded PSFs of SI-FLFM. (a). The simulated PSFs using the wave optics model. (b). The real recorded PSFs. Scale bar: 100 µm. Color coded depth: [-45,45] µm.

## System Synchronizatio

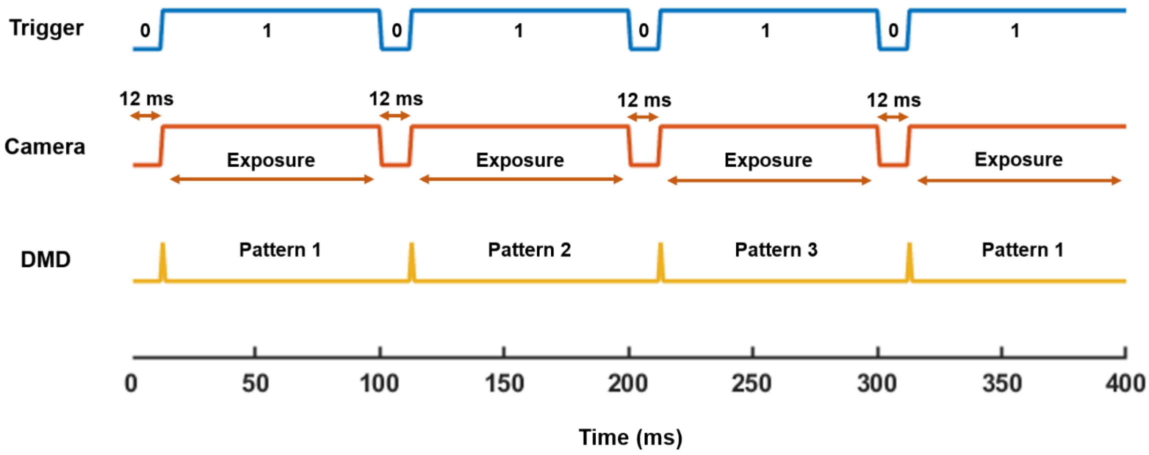


Fig. S2. Synchronization of the DMD and camera.

## Experimental Results

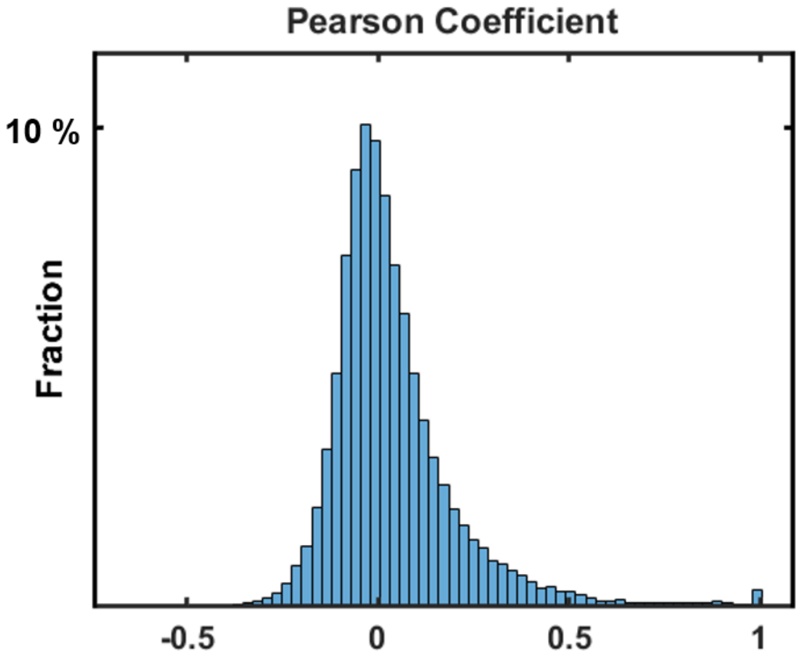


Fig. S3. Pearson coefficients of the neural activity traces in mouse brains *in vivo*.
